# Event-related potential measures of the intending process: Time course and related ERP components

**DOI:** 10.1186/1744-9081-6-15

**Published:** 2010-02-24

**Authors:** Guangheng Dong, Yanbo Hu, Hui Zhou

**Affiliations:** 1 Department of Psychology, Zhejiang Normal University, 688 of Yingbin Road, Jinhua City, Zhejiang Province, PR China; 2 Department of Psychology, Royal Holloway, University of London, Egham, UK

## Abstract

**Background:**

The intending process plays an important part to the successful completion of many daily activities. However, few researchers have paid attention to this issue. This study was set to investigate the time course and the electrophysiological evidence of the intending process with a cue-respond task.

**Methods:**

Event-related potentials (ERPs) were recorded while participants were performing different cued conditions (deceptive, truthful, and watch-only). The time course of intending process was analyzed through the different effect of the cue stimuli.

**Results:**

The P2 component, that appeared between 200 and 400 ms after the cue was onset, can be observed in the intended conditions (deceptive, truthful), but cannot be found in un-intended condition (watch-only). The mean amplitude in P2 between the truthful and deceptive conditions was consistent with previous studies. P2 was thought to be the reflection of the intention process.

**Conclusions:**

The results suggested that the intention process happened 200 to 400 ms after the cue stimuli was onset, and the P2 in the posterior scalp during this period could be a specific component that related with the process of intention.

## Background

The ability to plan ahead in time plays an important part to the successful completion of many daily activities. The mental construction that could direct future action was termed as 'intention'. The ability to prepare the execution of a behavioral response depends on the consideration of (A) changing task priorities and (B) the affordances of potential target stimuli that become present in the environment [1]. The time course of making intention and whether intentions exist irrevocably between the time of intent and the time of the action were of interest.

Localization studies found intention related activities in the parietal reach region (PRR), which were suggested to dominate the delay period in delayed movement tasks and to comprise a substantial component of the transient response [2,3]. These findings, along with additional anatomical and physiological evidences, suggested that different cortical areas within the posterior parietal cortex (PPC) could represent the preparations for different actions. Lesion studies also support the idea that the PPC is part of a specialized pathway for programming actions [4,5]. The anatomical features of the PPC suggest that different regions of PPC are functionally distinct [6]. A number of studies have suggested that the activations of the PPC are related to sensory stimuli, attention, and intentions to move [2].

There are fMRI [3] and lesion [5] studies provide important information about the brain areas that related with intention, the time course of brain activity using such techniques lacks of good temporal resolution and may not distinguish the fast change of the different stages of intending process. Event related potentials (ERPs) can be used in studying the temporal process of intention, as they provide an excellent and precise metric of the time course of neural activity [7,8]. Very few studies on intention process have been conducted with ERPs and therefore this is an exploratory study about intention with no specific predictions.

In order to distinguish the intention process from other mental processes, the cued deceptive paradigm was used. In the cued deceptive paradigm, participants were asked to press the relevant button that match with the truth answer in the truthful condition. In the deceptive condition, they were then told to try to hide the true answer and respond with the opposite one [9]. In our study, we added another cue condition: watch-only, in which participants were only asked to watch the target with no response required. Both deceptive and truthful cue stimuli were intention process, they all required participants to respond according to the stimuli. But the watch-only process was easy, because participants were not asked to react, there was no intention process. Thus, our hypothesis is that if there were ERPs that can be found in intention conditions (truthful, deceptive) and can't be observed in watch-only condition, that component may reflect the intention process.

In recent years, various approaches to psycho-physiological detection of deception have been developed. A lot of studies reported a reduction of ERP amplitudes in posterior region in lying vs. truth-telling conditions [10,11] and it was thought that this might reflect the inhibition of truthful responses while not requiring the either attention or processing resources [12,13]. Thus, we hypothesized that the similar ERP patterns may also be found between truthful and deceptive conditions.

Taking the feature of ERP waveforms and the amplitudes during the deceptive and truthful processes into consideration, we hope the feature of ERP waveforms in different conditions can help us find the time course that relates to ERPs during the intending process. In summary, the main focus of the study is to explore any specific ERP component that related to the intending process and also the time course of it.

## Methods

### Participants

Nineteen right-handed subjects participated in the experiments (10 female). Data from three subjects (1 male, 2 female) were discarded because of too many artifacts. The age ranges of the remaining subjects were 18.3 to 26.1 (mean age: 21.9 years). All of them had normal or corrected to normal vision and did not have any history of neurological disease. The experiment procedure was in accordance with the ethical principle of the 1964 Declaration of Helsinki (World Medical Organization).

### Materials

All of the cue stimuli were white with the size of 18 (width) × 54 (length) pixels and were presented on a black background using the E-Prime software (Psychology Software Tools Inc., Pittsburgh, Pennsylvania, USA). The target stimuli were facial pictures with no emotion expression (neutral faces), which were downloaded from the Internet randomly. All the stimuli pictures measured 320 × 240 pixels (when running the E-Prime software, the whole screen measures 640 × 480 pixels). There were 240 trials with 3 different valence conditions. The pictures showed the front part of the face and at least two thirds of the entire picture was used to present the face. All the pictures were colored gray, the background was black. To select the facial stimuli, 28 college students were asked to rate the valence of about 300 pictures (attractive vs. ugly) by self-report using a five-point rating scale before formal study. Based on their rating results, 60 attractive facial pictures (30 men, 30 women) and 60 ugly facial pictures (30 men, 30 women) were selected as stimuli materials in our study. All pictures were present in both blocks. So, there are 60 trials for each condition. To exclude personal opinions about pictures, responses that were not agreed with defaulted value were excluded from further analysis.

### Tasks and procedures

Subjects were seated approximately 80 cm away from a computer screen (Dell, 17-inch LCD monitor, 60-Hz refresh rate) with the horizontal and vertical visual angles below 5°. All subjects were instructed that they should keep fixating at the screen during all tasks.

In this study, each trial started with a small white cross (+) in the center of the screen for 250 ms followed by a cue word 'truthful', 'deceptive', or 'watch-only' that randomly presented in the centre of the screen for 1000 ms. After the cue, a target picture was presented for 1000 ms. The subjects were instructed to make the corresponding button-press response according to the cue that they were given prior to the target picture. In the truthful condition, subjects were required to make a truthful 'attractive (key 1) or ugly (key 2)' judgment about the pictures. In the deceptive condition, subjects were required to make a deceptive judgment about the pictures and give response opposite to the truth. In the 'watch-only' condition, participants were instructed to fixate on the pictures only.

In the truthful and the deceptive conditions, participants were asked to make proper responses according to the cue, therefore they would have to adjust their mood and prepare the coming response. However, this mental process was not a must in the watch-only condition. In the watch-only condition, participants only needed to watch the target, during which, they didn't need to activate their intending process. So we believed that the truthful or deceptive trials were intending process. The watch-only condition here viewed as control condition.

### ERP recording

High-density ERPs were recorded using a 128-channel geodesic sensor net (250 Hz, Electrical Geodesics Inc., (EGI) Eugene, Oregon, USA) coupled with a high input impedance amplifier. The EEG was continuously recorded with a sample rate of 250 Hz. Whenever possible, impedances were reduced to less than 50 KΩ (EGI default parameter) prior to recording with the vertical electrooculograms (EOG) recorded at the left orbital rim and the horizontal EOG recorded at the right orbital rim.

### ERP averaging

**Figure 1:**
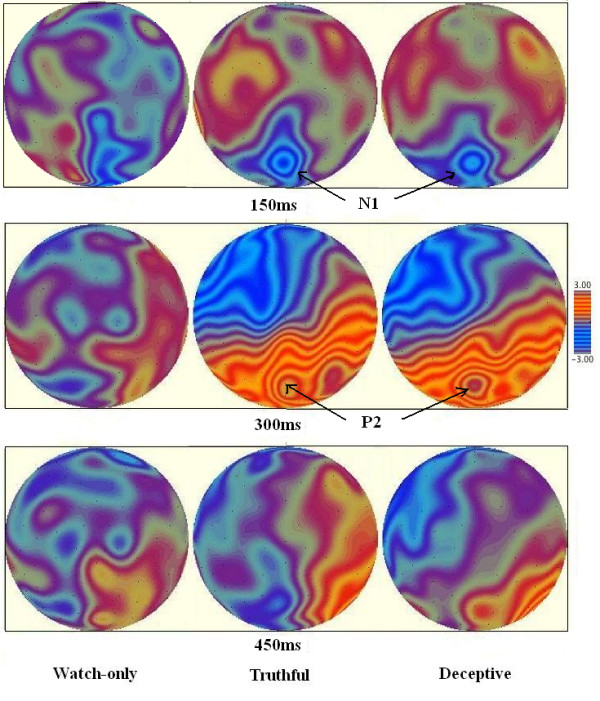
**Topographical maps**. Topographical maps in deceptive, truthful and watch-only conditions at 150 ms, 300 ms, 450 ms after the cue stimuli onset.

**Figure 2:**
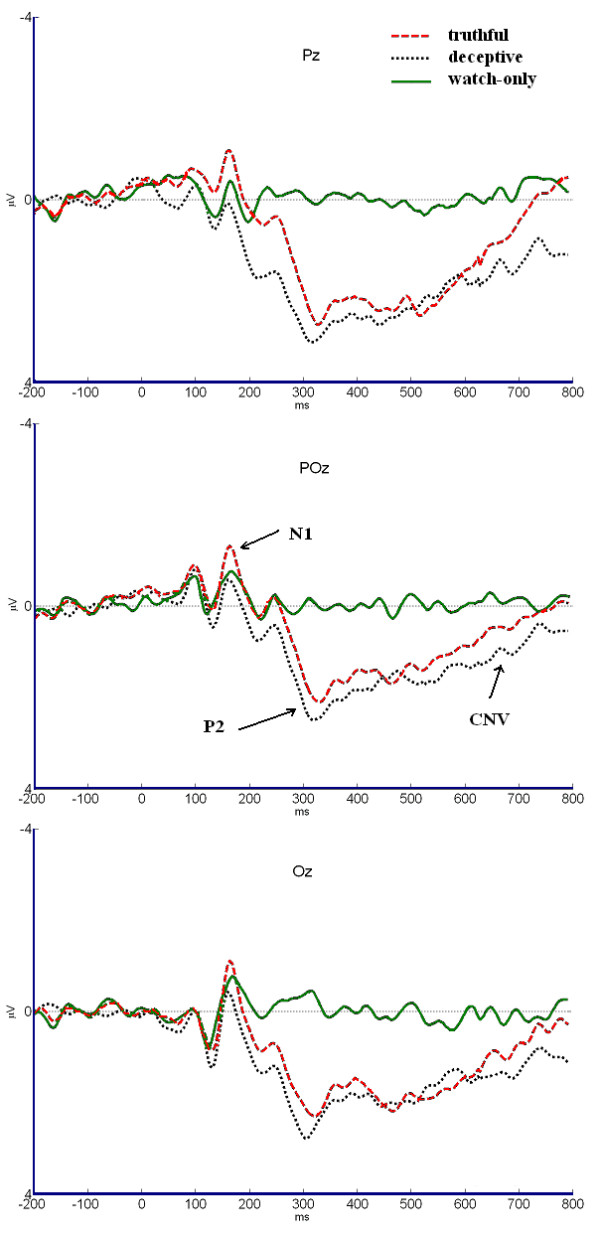
**Averaged ERP waveforms**. Averaged ERP waveforms in truthful, deceptive, and watch-only conditions at Pz, POz, and Oz electrode sites.

The data were analyzed offline with the software NetStation (Electrical Geodesics Inc., Eugene, Oregon, USA). Trials with incorrect responses and trials with EOG artifacts were excluded. The data were filtered with a band pass of 0.3-30 Hz. EEG activity for the correct response in each valence condition was overlapped and averaged separately. The ERP waveforms were time-locked to the onset of cue stimuli. The averaged epoch was 1000 ms, including a 200 ms pre-stimulus baseline. As indicated by the scalp topographic maps (Figure 1) and ERP's grand averaged waveforms at Pz, POz, Oz, the posterior region sites (Figure 2) showed prominent effects about 200-400 ms after the cue stimuli onset. Based on this, as well as on the previous localization studies on intention [2,3], we selected the following nine electrode sites for statistical analysis: Pz, P1, P2, POz, PO1, PO2, Oz, O1, O2, all of which located in posterior scalp. The mean amplitude (mean value of the selected time window) and the peak latency (from the time of the stimulus onset to the time of the peak activation of each component) of the N1 (150-200 ms), P2 (200-400 ms) and CNV (600-800 ms) were measured and analyzed. A repeated ANOVA was conducted for the amplitude and latency of each component. The ANOVA factors were valence conditions (truthful, deceptive and watch-only). Bonferroni correction was applied for multiple post-hoc comparisons.

## Results

### Behavioral performance

Responses that were too fast (less than 100 ms), too slow (more than 1000 ms), or incorrect were excluded from analysis. The mean reaction times for the truthful and the deceptive conditions in the study were 473.2 ms (*SD *= 238.3) and 487.4 ms (*SD *= 246.1) respectively. A repeated analysis showed no significant main effect of valence [*F*(1,15) = 1.127, *p *> 0.05]. Error rate was 0.082 (*SD *= 0.032) for the truthful condition and 0.091 (*SD *= 0.037) for the deceptive condition. No significant difference was found between the truthful and deceptive conditions [*F*(1,15) = 1.214, *p *> 0.05].

### ERP results

From ERP waveforms, we found that all of the three conditions elicited a distinct N1 over the posterior scalp regions. However, P2 and CNV were only found obviously in deceptive and truthful conditions. On the other hand, after 200 ms, the waveform was flat and no significant ERP waves were found in watch-only condition.

Between the time window of 150 and 200 ms, task type had a significant effect in mean amplitude [*F*(2,30) = 7.451, *p *< 0.05]. Post-hoc analysis showed that the mean amplitude was significantly more negative for deceptive items than that for truthful [*F*(1,15) = 5.132, *p *< 0.05] and watch-only [*F*(1,15) = 4.711, *p *< 0.05] items. In addition, no significant main effect was found between truthful and watch only items [*F*(1,15) = 1.057, *p *> 0.05].

Task type had a significant main effect in the mean amplitude during P2 [*F*(2,30) = 24.446, *p *< 0.01]. Post-hoc analysis showed that both the deceptive [*F*(1,15) = 19.301, *p *< 0.01] and the truthful [*F*(1,15) = 15.963, *p *< 0.01] condition showed more negative mean amplitude of P2 than watch-only condition did. In addition, deceptive items showed less negative mean amplitude of P2 than that of the truthful items [*F*(1,15) = 5.103, *p *< 0.05].

In the mean amplitude during CNV (600-800 ms), task type had a significant main effect in mean amplitude [*F*(2,30) = 12.197, *p *< 0.01]. Post-hoc analysis showed more negative mean amplitude the deceptive condition than in truthful condition [*F*(1,15) = 5.753, *p *< 0.05].

No significant difference in peak latency was observed between these three conditions in N1 [*F*(2,30) = 0.773, *p *> 0.05], P2 [*F*(2,30) = 1.042, *p *> 0.05] and CNV [*F*(2,30) = 0.190, *p *> 0.05] (Table 1).

**Table 1:** The mean amplitudes of N1, P2, and CNV components collapsed across all selected electrode sites in different conditions

	Truthful		Deceptive		Watch-only	
	*M*	*SD*	*M*	*SD*	*M*	*SD*
N1	-0.52	0.13	-0.71	0.16	-0.55	0.15
P2	2.11	0.43	1.69	0.26	0.11	0.02
CNV	1.07	0.19	0.94	0.18	-0.16	0.05

*M*, arithmetic mean; *SD*, standard deviation

## Discussions

The present study explored the electrophysiological evidence of the process of intending to deceive with a cue-response paradigm. ERP results in N1 (150-200 ms) showed that the deceptive items elicited a more negative ERP deflection than that of the truthful and watch-only items. The truthful items elicited a more positive ERP deflection than that of the deceptive items in P2 (200-400 ms). Additionally, the deceptive items elicited a more negative CNV (600-800 ms) than that of the truthful items.

All of the three conditions elicited significant N1 between 150 and 200 ms, which suggested that N1 was associated with the common ground of these three conditions. The N1 may reflect the different early lexical semantic access of the cue stimuli for the three conditions. Studies demonstrated that the brain could distinguish different target words at about 200 ms after target onset [14], which could indicate that a great amount of lexical information including lexical semantics, should have been processed within the first 200 ms [15]. The difference between these three valences brought more support evidence. As the cue words were emotional (e.g., the cue 'deceptive' was a negative word, the 'truthful' was a positive word and 'watch-only' was neutral), the different valence of affective words may bring different emotion priming [16]. Studies on the valence of emotionally affective words have shown that N1 was sensitive to emotion valence [17]. Scott et, al. used affective words as stimuli material and found that the N1 (135-180 ms) showed a significant emotion in posterior scalp. And their ERP data suggested an early identification of the emotional tone of words leading to differential processing, specifically, negative words which seemed to attract additional cognitive resources [18]. Therefore, we suggested that participants could distinguish the lexical semantic of different stimuli at about 150-200 ms after the stimuli onset. In addition, the negative brain response peaking between 150 and 200 ms could be associated with the processing of different cue words.

Another important ERP component was P2 in the posterior scalp, which can be observed between 200 and 400 ms. The distribution of P2 was consistent with previous fMRI and anatomical studies about intention which also found activation in posterior regions (PRR, PPC) during intention process [2,4]. In our study, P2 can obviously be observed during the truthful and deceptive conditions, but not in watch-only condition. In the watch-only condition, the waveform after N1 was flat and no significant ERPs were found. The features of the task could explain the ERP features. In the truthful and the deceptive conditions, participants were asked to make proper responses according to the cue, therefore they would have to adjust their mood and prepare the coming response. However, this mental process was not a must in the watch-only condition. In the watch-only condition, participants only needed to watch the target, during which, they don't need to activate their intending process. Comparative studies between the truthful and deceptive conditions showed that the truthful condition elicited higher positive deflection than the deceptive condition did during deceptive process. Previous ERP studies about deception have reported a reduction in LPC amplitude in lying versus truth-telling conditions [10]. Johnson et al. suggested that decreases in LPC amplitude might reflect the inhibition of the truthful responses, taking the attention and/or the processing resources away from the primary task of responding truthfully [9]. Although the ERP component P2 in present study was not the traditional LPC in the target-locked procedures, the features between the truthful and deceptive conditions that were found in P2 were similar to LPC: less activation for the deceptive condition than that for the truthful condition. So, the P2 amplitude decrements might have reflected the processing resources were drawn away from the primary evaluation to make deceptive intention. Taken the waveform features and the comparison between deceptive and truthful conditions into consideration, we suggest that P2 in posterior scalp during 200 and 400 ms is an ERP component associated with intention process.

Another ERP component was the CNV on the posterior scalp. During this period, participants made their decisions and wait for the targets to appear. Using visual and tactile stimuli in the supra-second range, Macar and Vidal reported that the CNV amplitude at electrode CPz peaked at the end of the memorized standard, even when the current test duration went beyond [19]. Larger CNV amplitude would reflect more accumulated pulses that leading to a longer perceived duration. This result has been reproduced with auditory filled intervals in the sub-second range [20]. In our study, the deceptive items elicited higher mean amplitude than the truthful items did, which suggested that the deceptive process required more cognitive endeavors leading to a longer perceived duration.

## Limitations

Some limitations of present study should be noted. Firstly, during the intending process, it remains difficult to control a participant's strategy use. Future studies should therefore determine other methods to control this issue. Secondly, the waiting period lasted for 1000 ms during this study, what would happen if we prolong or shorten the presentation time of the cue stimuli? These hypotheses should be tested in forthcoming studies.

## Conclusions

From what we have discussed above, we could divide the time course of making intention into 3 steps: First, the lexical semantic understanding progress, which happened between 150-200 ms after the cue stimuli onset; second, the intention process, which happened between 200-400 ms after the stimuli onset and third, the waiting process, which happened at about 600 ms after the stimuli onset. The time course of intention took placed between 200 and 400 ms after the stimuli onset. The P2 in posterior scalp during this period could be a component related with intention process.

## List of abbreviations

ANOVA: Analyses of variance; CNV: Contingent negative variation; EEG: electroencephalogram; EOG: electrooculograms; ERP: Event-Related potential; fMRI: Functional magnetic resourcing imaging; LPC: Later positive component; PPC: Posterior parietal cortex; PRR: Parietal reach region; SD: Standard deviation.

## Competing interests

The authors declare that they have no competing interests.

## Authors' contributions

GD carried out the study. YH participated in the design of the study. HZ performed the statistical analysis. All authors read and approved the final manuscript.
